# Anti‐Angiogenic Agents for Advanced Hepatocellular Carcinoma Induce Liver Atrophy

**DOI:** 10.1002/cam4.71066

**Published:** 2025-07-25

**Authors:** Taijiro Wake, Tomoharu Yamada, Ryosuke Tateishi, Makoto Moriyama, Yuki Matsushita, Kazuya Okushin, Takuma Nakatsuka, Masaya Sato, Tatsuya Minami, Yotaro Kudo, Mitsuhiro Fujishiro

**Affiliations:** ^1^ Department of Gastroenterology, Graduate School of Medicine The University of Tokyo Tokyo Japan

**Keywords:** anti‐programmed cell death ligand 1, atezolizumab, bevacizumab, Child‐Pugh score, computed tomography, immune checkpoint inhibitor, lenvatinib, liver volume, magnetic resonance imaging, vascular endothelial growth factor

## Abstract

**Aim:**

Systemic therapy for advanced hepatocellular carcinoma (HCC) includes multi‐kinase inhibitors with anti‐vascular endothelial growth factor (VEGF) activity and anti‐VEGF monoclonal antibodies in combination with immune checkpoint inhibitors. This study aimed to investigate and compare the chronological changes in liver volume between patients who received atezolizumab plus bevacizumab (Atezo/Bev) and those who received lenvatinib.

**Methods:**

We enrolled patients who received initial treatment with either Atezo/Bev or lenvatinib for advanced HCC between October 2018 and May 2023. Patients underwent periodic computed tomography (CT) or magnetic resonance imaging (MRI) to evaluate systemic therapy effects. Patients with portal vein thrombosis or prior liver resection/transplantation were excluded. Liver volume was measured at baseline and at 8 and 16 weeks after the initiation of treatment using commercially available software. Liver volume at each time point was expressed as a proportion relative to baseline. A linear regression analysis was used to analyze the chronological changes in liver volume.

**Results:**

Seventy‐three patients (40 in the Atezo/Bev group and 33 in the lenvatinib group) were included in this retrospective study. Liver volume decreased in 54 patients (74%) at week 8; the average volume relative to baseline was 0.92 (95% confidence interval: 0.90–0.94, *p* < 0.01). Liver volume decreased in patients with both shrinking and enlarged tumors. Multivariate analysis indicates that the decrease in nontumoral liver volume was more significant in the lenvatinib group than in the Atezo/Bev group (*p* = 0.04).

**Conclusions:**

Anti‐angiogenic therapy for advanced HCC can lead to liver atrophy.

**Abbreviations**
AFP: alpha‐fetoprotein
Atezo/Bev: atezolizumab plus bevacizumab
BCLC: Barcelona Clinic Liver Cancer
CT: computed tomography
CTCAE: Common Terminology Criteria for Adverse Events
FGF: fibroblast growth factor
Gd‐EOB‐DTPA: gadolinium ethoxybenzyl diethylenetriamine pentaacetic acid
HCC: hepatocellular carcinoma
IQR: interquartile range
LEN: lenvatinib
MRI: magnetic resonance imaging
PD‐L1: programmed cell‐death ligand 1
RDI: relative dose intensity
RECIST: Response Evaluation Criteria in Solid Tumors
RFA: radiofrequency ablation
TACE: transarterial chemoembolization
VEGF: vascular endothelial growth factor

## 1 Introduction

Primary liver cancer is a significant global health burden and ranks as the sixth most diagnosed cancer and the third leading cause of cancer‐related deaths worldwide [1]. Hepatocellular carcinoma (HCC) is the most common type of primary liver cancer [2]. HCC is frequently diagnosed at advanced stages, at which systemic therapy is commonly indicated [3]. The current regimens include multi‐kinase inhibitors, humanized anti‐vascular endothelial growth factor (VEGF) monoclonal antibodies, antiprogrammed cell death ligand 1 (PD‐L1) inhibitors, and combinations thereof [4, 5, 6, 7]. Many of these agents share the common feature of inhibiting the VEGF pathway, which is associated with cancer cell proliferation and angiogenesis. Although the efficacy of such angiogenic therapies has been demonstrated, blocking the VEGF pathway may inhibit liver regeneration because neovascularization is primarily regulated by VEGF [8, 9]. In fact, patients treated with sorafenib, a multi‐kinase inhibitor that targets the VEGF receptor (VEGFR), experienced impaired liver regeneration after hepatectomy for HCC [10, 11]. In addition, one study reported that bevacizumab, a humanized monoclonal antibody against VEGF, impaired liver hypertrophy induced by portal vein occlusion prior to major hepatectomy [12].

In contrast, two reports suggested that preoperative administration of bevacizumab did not affect liver regeneration after hepatectomy for colorectal cancer [13, 14]. These paradoxical results may be caused by differences in clinical settings and the absence of chronic liver disease in patients with liver metastases from colorectal cancer. Alternatively, the effects of multi‐kinase inhibitors and antibodies against VEGF on liver regeneration may differ. The aim of this study was to measure the liver volume of patients receiving anti‐angiogenic agents for advanced HCC and to investigate the changes over time.

## 2 Materials and Methods

### 2.1 Patient Enrollment

Systemic therapies are indicated for patients with HCC in whom surgical resection, liver transplantation, and local therapies, such as radiofrequency ablation (RFA) and transarterial chemoembolization (TACE), are not applicable or unsuitable according to the clinical practice guidelines for liver cancer published by the Japan Society of Hepatology [15]. We enrolled patients with HCC who were treated with either atezolizumab and bevacizumab combination therapy (Atezo/Bev) or lenvatinib as first‐line therapy between October 2018 and May 2023. Patients with portal tumor invasion, those who had undergone hepatic resection within six months before the start of systemic therapy, and those who had undergone liver transplantation were excluded because these conditions significantly affect liver regeneration [16, 17].

### 2.2 Atezo/Bev and Lenvatinib Treatment

In the Atezo/Bev group, patients were given atezolizumab (1200 mg, intravenously) and bevacizumab (15 mg/kg, intravenously) in a single cycle and received treatment every three weeks. Patients visited the outpatient clinic every three weeks, and clinical examinations were conducted before the administration of Atezo/Bev. Lenvatinib was orally administered at a dose of 12 mg/day for patients weighing 60 kg or more and 8 mg/day for patients weighing less than 60 kg. Both groups underwent an efficacy assessment based on version 1.1 of the Response Evaluation Criteria in Solid Tumors (RECIST) guidelines [18], and decisions regarding the continuation or modification of treatment were made by the attending physician based on this assessment. Adverse events were evaluated based on version 5.0 of the Common Terminology Criteria for Adverse Events (CTCAE). Treatment was discontinued or postponed if grade 3 or higher adverse events were observed.

### 2.3 Data Collection

The baseline characteristics of the enrolled patients, including age, sex, etiology, Child‐Pugh score, extrahepatic metastasis, Barcelona Clinic Liver Cancer (BCLC) classification, albumin, total bilirubin, platelet count, prothrombin time, tumor markers, liver volume, and tumor volume, were obtained prior to systemic therapy. The relative dose intensity (RDI) was calculated as the ratio of the administered dose to the standard dose [19].

### 2.4 Liver Volume Measurement

In our hospital, patients receiving Atezo/Bev are scheduled for contrast‐enhanced computed tomography (CT) scans every three cycles, whereas patients receiving lenvatinib are scheduled for contrast‐enhanced CT scans every eight weeks. Therefore, the imaging assessments in both groups were nearly matched at 8 (first imaging assessment) and 16 weeks (second imaging assessment) after the initiation of treatment for HCC (Figure S1). Patients ineligible for contrast‐enhanced CT scans because of renal impairment or iodine allergy were assessed using gadolinium ethoxybenzyl diethylenetriamine pentaacetic acid (Gd‐EOB‐DTPA) magnetic resonance imaging (MRI).

Nontumoral and tumoral liver volumes were measured using the commercially available software programs Ziostation 2 (Ziosoft Inc., Tokyo, Japan) for CT images and SYNAPSE VINCENT (Fujifilm Co. Ltd., Tokyo, Japan) for MRI images.

Ziostation2 is a software system that underwent internal accuracy management by the manufacturer and received regulatory approval in Japan based on these data (Approval number: 223ABBZX00032000). Validation data were submitted to the Pharmaceuticals and Medical Devices Agency (PMDA) as part of the approval process; however, these data are not publicly available. VINCENT has been reported in a previous study to provide highly accurate volumetric calculations when compared with actual resected liver specimens [20]. The nontumoral portion was defined as the liver parenchyma enhanced in the portal venous phase of contrast‐enhanced CT, whereas the tumor was defined as the area where the contrast agent was washed out. In Gd‐EOB‐DTPA‐enhanced MRI, the hepatobiliary phase was used for the same purpose. Post‐RFA areas, cysts, and regions with lipiodol deposition were excluded from liver volume measurements.

### 2.5 Statistical Analysis

Data are presented as means and standard deviations or as medians and interquartile ranges (IQR) for quantitative variables and as numbers and percentages for qualitative variables. The primary endpoint was the difference in liver volume from baseline at 8 weeks. Subgroup analyses were performed according to treatment regimens and responses at week 8. A linear regression analysis was conducted to assess the factors contributing to changes in liver volume. The dependent variable was the change in nontumoral liver volume, standardized according to the following formula [21]:

$$\text{Standard liver volume}\;\left({\mathrm{cm}}^{3}\right)=706.2\times \text{body surface area}\;\left(m^{2}\right)+2.4$$

The independent variables were age, sex, treatment regimen, etiology, albumin level, total bilirubin level, platelet count, prothrombin time, and alpha‐fetoprotein (AFP).

All statistical analyses were performed using R version 4.2.2 (R Development Core Team, Vienna, Austria), with *p*‐values < 0.05 considered statistically significant.

## 3 Results

### 3.1 Patient Profiles

**FIGURE 1:**
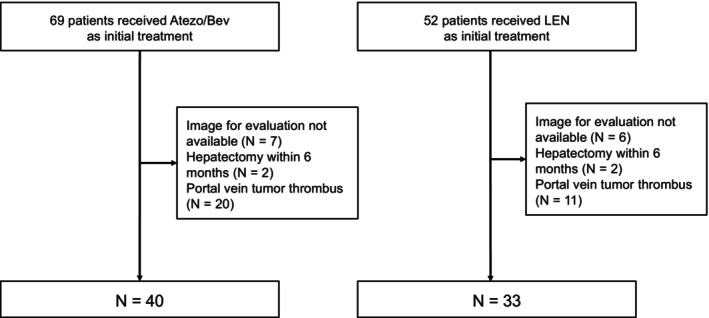
Patient flowchart. Atezo/Bev, atezolizumab plus bevacizumab; LEN, lenvatinib.

Among the 121 patients with HCC who underwent first‐line systemic therapy with either Atezo/Bev or lenvatinib during the study period, 73 patients (40 treated with Atezo/Bev and 33 treated with lenvatinib) were included in this study (Figure 1).

The baseline patient characteristics are shown in Table 1. The mean age was 71.9 years (range, 55–87 years) and 71.7 years (range, 45–89 years) in the Atezo/Bev and lenvatinib groups, respectively. The lenvatinib group was more likely to have better Child‐Pugh scores (*p* = 0.09) and lower platelet counts (*p* = 0.08) than the Atezo/Bev group. The RDI was significantly higher in the Atezo/Bev group (*p* < 0.001). In addition, the median nontumoral liver volume at baseline was 1243 cm^3^ (IQR, 1083–1548 cm^3^) in the Atezo/Bev group and 1259 cm^3^ (IQR, 1141–1448 cm^3^) in the lenvatinib group. The adverse events are shown in Table S1. Proteinuria (27.5% and 36.4%, respectively) and fatigue (27.5% and 39.4%, respectively) were observed in both the Atezo/Bev and lenvatinib groups, with a higher incidence in the lenvatinib group.

**TABLE 1:** Baseline characteristics of patients (*N* = 73).^a^

Characteristic	Atezo/Bev group *N* = 40	LEN group *N* = 33	*p*
Age, mean ± SD	71.9 ± 9.0	72.2 ± 9.5	0.90
Male sex, *n* (%)	32 (80)	30 (91)	0.33
Viral infection, *n* (%)			
HBsAg positive	6 (15)	3 (9)	0.50
Anti‐HCVAb positive	8 (20)	11 (33)	0.28
Both positive	1 (2.5)	0 (0)	0.84
Both negative	27 (67.5)	19 (58)	0.47
Child‐Pugh, *n* (%)			
Class 5A	22 (55)	24 (73)	0.09
Class 6A	14 (35)	8 (24)	
Class 7B	4 (10)	1 (3)	
Extrahepatic spread, *n* (%)	8 (20)	7 (21)	> 0.99
BCLC stage, *n* (%)			
Early	0 (0)	3 (9)	0.28
Intermediate	26 (65)	20 (61)	
Advanced	14 (35)	10 (30)	
Albumin, g/dL	3.8 (3.4–4.0)	3.7 (3.6–4.0)	0.71
Total bilirubin, mg/dL	0.8 (0.6–1.0)	0.7 (0.6–1.0)	0.78
Platelet count, ×10^4^/μL	16.7 (12.3–23.0)	13.1 (9.6–17.5)	0.08
Prothrombin time, %	92 (85.2–98.4)	100 (97–100)	0.002
AFP > 400 ng/mL, (%)	15 (38)	8 (24)	0.34
DCP, mAU/mL	309 (77–1667)	349 (44–2206)	0.72
Prior locoregional therapy, *n* (%)			
Surgery	18 (45)	14 (42)	1
RFA	24 (60)	20 (61)	1
TACE	20 (50)	22 (67)	0.23
RDI, %	100 (100–100)^b^	67.9 (62.2–91.4)	< 0.001
Liver volume, cm^3^	1243 (1083–1548)	1259 (1141–1448)	0.87
Tumor volume, cm^3^	28 (6–132)	21 (7–53)	0.49

Abbreviations: AFP, alpha‐fetoprotein; Atezo/Bev, atezolizumab plus bevacizumab; BCLC, Barcelona Clinic Liver Cancer; DCP, des‐γ‐carboxy prothrombin; HBsAg, hepatitis B surface antigen; HCVAb, hepatitis C virus antibody; LEN, lenvatinib; RDI, relative dose intensity; RFA, radiofrequency ablation; SD, standard deviation; TACE, transarterial chemoembolization.

^a^ Values are expressed as median (25th‐75th percentiles) or *n* (%).

^b^ Out of 40 patients, 31 patients received a 100% dosage.

### 3.2 Change in Liver Volume

**FIGURE 2:**
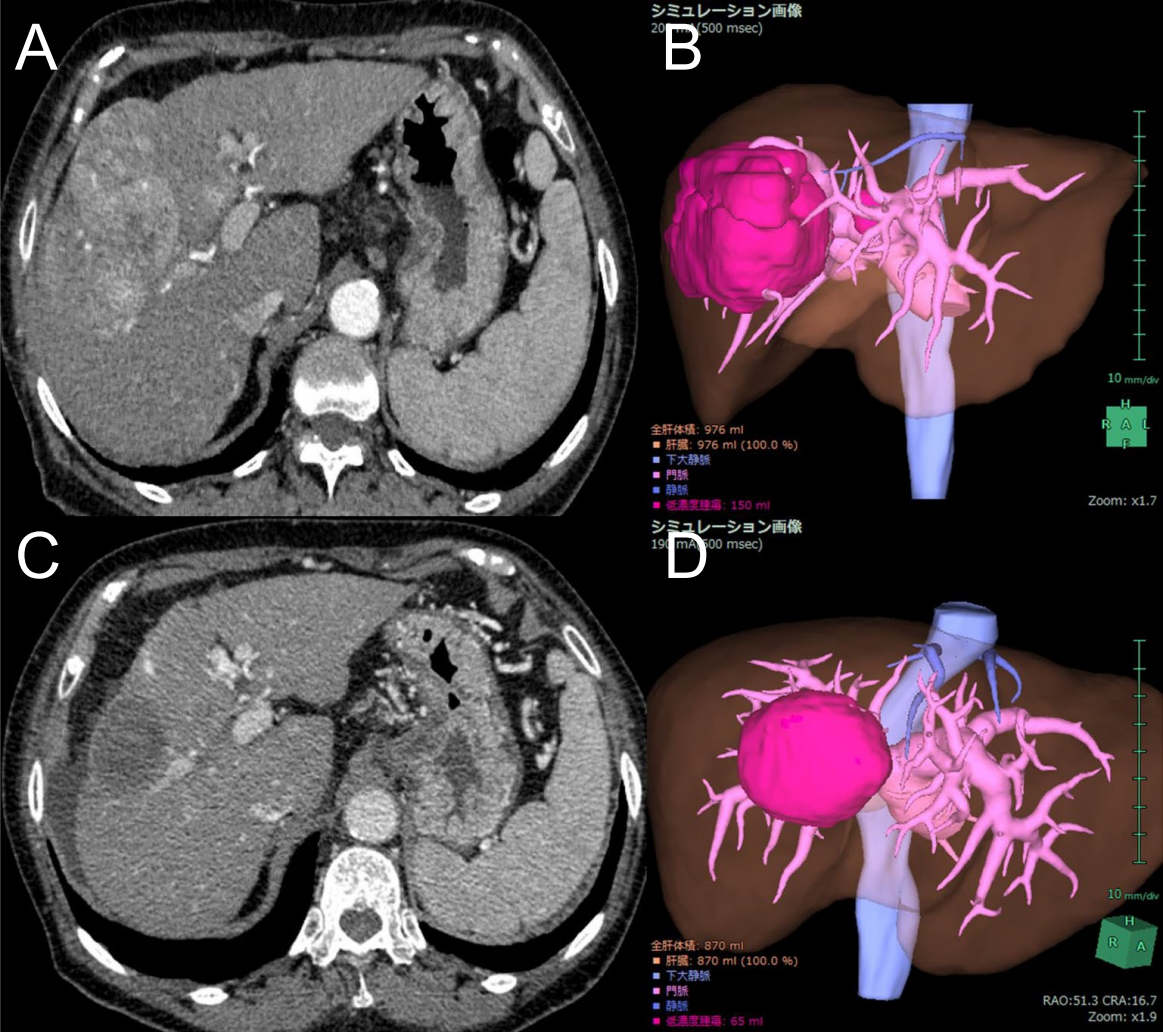
A case with significant liver atrophy after treatment with Atezo/Bev. (A) The patient had a hepatocellular carcinoma in segment 5/8. (B) The nontumoral liver volume (light brown) was 976 cm^3^, and the tumoral volume (pink) was 150 cm^3^. (C) After three courses of treatment, the target tumor shrank and exhibited reduced enhancement. Liver atrophy with ascites was also observed. (D) The nontumoral and tumoral liver volumes decreased to 870 cm^3^ (−11%) and 65 cm^3^ (−57%), respectively. Atezo/Bev, atezolizumab plus bevacizumab.

**FIGURE 3:**
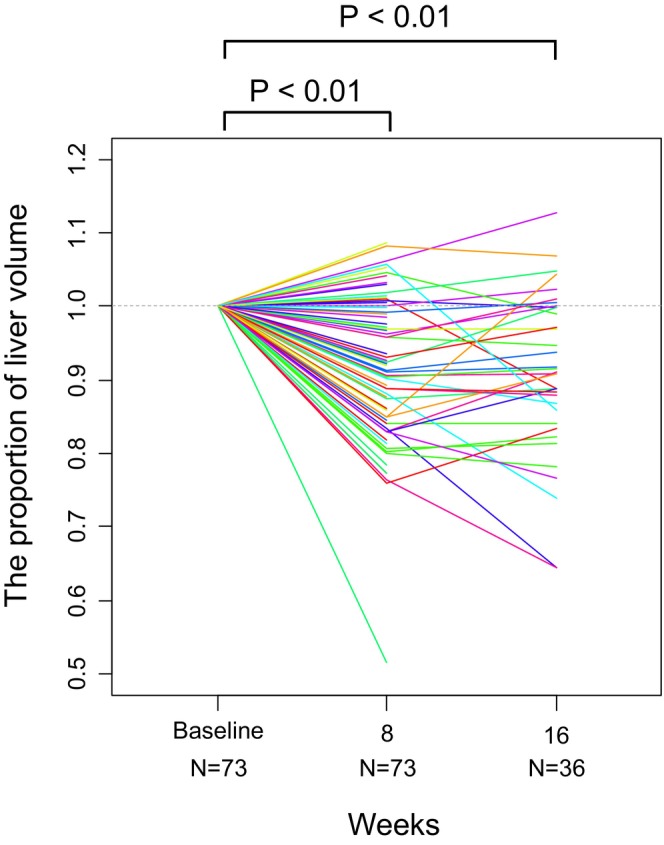
Line graph indicating changes in liver volume in patients who received systemic treatment. Each line in a different color indicates an individual patient. The liver volume decreased in 54 out of 73 patients (74%) at week 8. The average volume relative to baseline was 0.92 (95% CI: 0.90–0.94, *p* < 0.01) at week 8 and 0.91 (95% CI: 0.88–0.93, *p* < 0.01) at week 16. CI, confidence interval.

**FIGURE 4:**
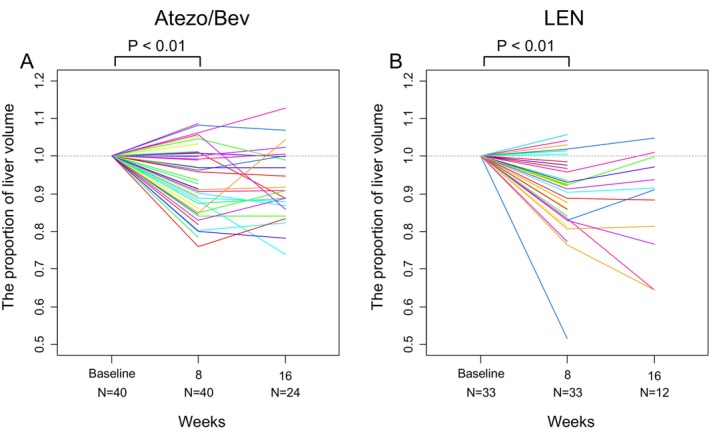
Subgroup analysis according to treatment regimen. Each line in a different color indicates an individual patient. At week 8, the liver volume decreased in both the (A) Atezo/Bev group (mean, 0.94; 95% CI, 0.91–0.97; *p* < 0.01) and the (B) lenvatinib group (mean, 0.90; 95% CI, 0.86–0.94; *p* < 0.01). Atezo/Bev, atezolizumab plus bevacizumab; LEN, lenvatinib.

The nontumoral liver volume at 8 weeks compared to the baseline was 0.92 on average (95% confidence interval [CI], 0.90–0.94; *p* < 0.01). The nontumoral liver volume decreased in 54 patients (74%). A representative case of reduced liver volume is shown in Figure 2. The liver volume at week 16 was available for 36 patients and was 0.91 times the baseline on average (95% CI, 0.88–0.93; *p* < 0.01) (Figure 3). Subgroup analysis by treatment regimen also showed that the liver volume at 8 weeks decreased in 28 patients (70%) in the Atezo/Bev group and 26 patients (79%) in the lenvatinib group. When analyzed across all patients, the liver volume at 8 weeks still significantly decreased in both the Atezo/Bev (mean, 0.94; 95% CI, 0.91–0.97; *p* < 0.01) and lenvatinib groups (mean, 0.90; 95% CI, 0.86–0.94; *p* < 0.01) (Figure 4A,B).

**FIGURE 5:**
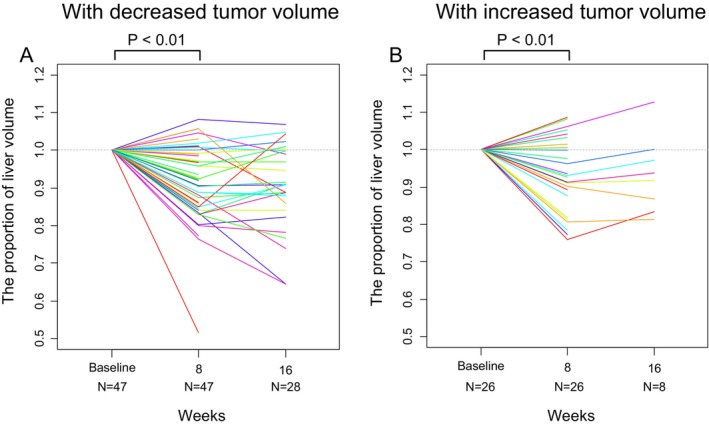
Subgroup analysis of cases in which tumor volume decreased or increased at week 8. The nontumoral liver volume reduced relative to baseline in both patients with (A) decreased tumor volume (mean, 0.91; 95% CI, 0.88–0.94; *p* < 0.01) and those with (B) increased tumor volume (mean, 0.93; 95% CI, 0.89–0.97; *p* < 0.01).

Another subgroup analysis based on the change in tumor size showed that the nontumoral liver volume decreased relative to baseline in both patients with reduced tumor volume (mean, 0.91; 95% CI, 0.88–0.94; *p* < 0.01) and those without (mean, 0.93; 95% CI, 0.89–0.97; *p* < 0.01) (Figure 5A,B). The liver volume decreased at week 8 in 36 out of 47 patients (77%) and in 18 out of 26 patients (69%), respectively.

### 3.3 Factors Contributing to the Change in Liver Volume

Linear regression analysis was performed to identify factors related to changes in liver volume (Table 2). Multivariate analysis indicates that the decrease in nontumoral liver volume was more significant in the lenvatinib group than in the Atezo/Bev group (further decrease of 0.067 in terms of standard liver volume; 95% CI, −0.13 to −0.003; *p* = 0.04).

**TABLE 2:** Linear regression analysis of change in liver volume (*N* = 73).^a^

Variable	Univariate			Multivariate		
	*β*	95% CI	*p*	*β*	95% CI	*p*
Age, per year	0.001	−0.002–0.004	0.38	0.002	−0.001–0.005	0.26
Male vs. female	−0.05	−0.13–0.03	0.24	−0.03	−0.11–0.06	0.52
LEN vs. Atezo/Bev	−0.04	−0.1–0.01	0.12	−0.067	−0.13 to −0.004	0.04
HBsAg positive	0.04	−0.04–0.13	0.30	0.056	−0.03–0.145	0.22
Anti‐HCVAb positive	0.008	−0.06–0.07	0.81	−0.007	−0.07–0.06	0.83
Albumin, per 1.0 g/dL	0.04	−0.03–0.11	0.25	0.024	−0.05–0.10	0.55
Total bilirubin, per 1 mg/dL	−0.08	−0.16–0.004	0.06	−0.07	−0.16–0.017	0.11
Platelet count, per 1 × 10^4^/μL	0.0004	−0.003–0.004	0.80	−0.002	−0.006–0.002	0.30
Prothrombin time, %	0.002	−0.001–0.006	0.13	0.004	−0.0003–0.008	0.07
AFP > 400 ng/mL	0.01	−0.05–0.07	0.70	0.02	−0.05–0.08	0.57

Abbreviations: AFP, alpha‐fetoprotein; Atezo/Bev, atezolizumab plus bevacizumab; CI, confidence interval; HBsAg, hepatitis B virus surface antigen; HCV, hepatitis C virus antibody; LEN, lenvatinib.

^a^ All variables were obtained before the initial treatment. Liver volume was standardized based on the estimated total liver volume. The dependent variable was the difference between liver volume at 8 weeks and that at baseline. The positive value of the regression coefficient (*β*) indicates increased liver volume.

## 4 Discussion

This study revealed that in patients with unresectable HCC, the nontumoral liver volume decreased at week 8 compared to baseline following treatment with either Atezo/Bev or lenvatinib. These results are consistent with those of previous reports showing that VEGF blockade impairs liver regeneration. The findings of this study highlight an important issue, as decreased liver function often prevents the continuation of treatment in clinical practice.

The liver can regenerate after surgery, transplantation, or damage such as acute liver failure. Liver regeneration involves angiogenesis [8], and controversy persists regarding whether VEGF pathway inhibitors, which block angiogenesis, also inhibit liver regeneration [12, 13, 14, 22, 23, 24]. Although previous studies have reported that preoperative administration of bevacizumab does not affect liver regeneration in hepatic resection for liver metastases from colorectal cancer [13, 14], our study suggests that the presence of underlying chronic liver disease may make the effect more apparent. Among the VEGF family members, VEGF‐A is strongly upregulated in the regenerating liver. Studies have shown that VEGF‐A stimulates sinusoidal endothelial cells to release hepatocyte growth factor (HGF), which in turn promotes the proliferation of hepatocytes [25]. Despite their different molecular characteristics, the monoclonal antibody bevacizumab (anti‐VEGF antibody) and the small‐molecule compound lenvatinib (a VEGFR inhibitor) both reduced liver volume. This finding suggests that VEGF plays a crucial role. One possible mechanism for the reduction in liver volume is that the drug reduces blood flow to the liver. Several members of the fibroblast growth factor (FGF) family are also expressed in the regenerating liver, and FGFs have been reported to have mitogenic effects on hepatocytes [25, 26]. In the mouse model, lenvatinib induced a greater reduction in CD31‐positive microvessels compared to VEGF inhibitors, suggesting a more potent anti‐angiogenic effect [27]. Whereas bevacizumab selectively targets VEGF‐A, lenvatinib inhibits VEGF receptors, FGF receptors 1–4, and other receptor tyrosine kinases. This broader spectrum of receptor inhibition may lead to a more profound suppression of hepatocyte proliferation and liver regeneration, potentially contributing to the more marked reduction in liver volume observed in the lenvatinib group compared to the Atezo/Bev group. When VEGF inhibition is associated with liver atrophy, combination immunotherapy regimens that do not involve VEGF inhibitors may become a treatment option for patients at high risk for liver atrophy. Additionally, even in later treatment lines, further development of non‐VEGF‐targeting agents, such as metronomic capecitabine, is warranted [28].

Compared with week 8, the number of evaluable cases at week 16 decreased due to factors such as treatment discontinuation from disease progression, adverse events, or conversion surgeries. In analyzing the week 16 data, the possibility of selection bias should be considered, as patients without liver volume measurements at this time point may have been in worse clinical condition. However, given that the liver volume at week 16 relative to baseline averaged 0.91 (95% confidence interval, 0.88–0.93; *p* < 0.01), it is likely that liver volume remained reduced on average in patients with available measurements. These findings suggest that liver atrophy occurs early after treatment initiation, and early imaging evaluation is therefore useful.

There are close relationships between the liver functional reserve and the liver volume. When we evaluated the change in albumin levels in those whose liver volume decreased at week 8, the albumin level significantly decreased by 0.35 g/dL on average. In addition, liver atrophy contributes to increased portal pressure because of decreased liver perfusion volume. Hatanaka et al. reported that the splenic volume increased in patients with HCC treated with lenvatinib [29]. In addition, Hidaka et al. investigated portal hemodynamics using Doppler ultrasonography in patients with HCC treated with lenvatinib and reported a significant decrease in portal venous flow velocity [30]. Gastroesophageal variceal rupture is a common adverse event in patients treated with anti‐angiogenic agents [31] and can lead to a vicious cycle in which decreased portal blood flow causes further hepatic atrophy. The ascites in the representative case in Figure 2 might be caused by the decreased albumin value and increased portal pressure.

This study had several limitations. First, untreated patients were not included as controls, suggesting that the reduction in nontumoral volume may be a natural course of advanced HCC. To address this issue, we performed a subgroup analysis to investigate the effect of tumor growth or shrinkage on changes in nontumoral volume. The results indicated that the decrease in nontumoral volume was unrelated to the treatment response. Second, this was a single‐institution study, which may limit its generalizability. To counter this limitation, we treated patients according to a standardized protocol, and scheduled images were obtained at 8 weeks in all cases.

In conclusion, antiangiogenic therapy for advanced HCC may induce liver atrophy. Based on these findings, we believe that clinicians should assess both tumor size and nontumoral liver volume during imaging evaluations. Further research is needed to determine optimal treatment strategies, including treatment selection, dose modification, and timing of treatment changes based on liver parenchymal volume.

## Author Contributions

**Taijiro Wake:** writing – original draft (equal). **Tomoharu Yamada:** writing – original draft (equal). **Ryosuke Tateishi:** conceptualization (equal). **Makoto Moriyama:** resources (supporting). **Yuki Matsushita:** resources (supporting). **Kazuya Okushin:** resources (supporting). **Takuma Nakatsuka:** resources (supporting). **Masaya Sato:** resources (supporting). **Tatsuya Minami:** resources (supporting). **Yotaro Kudo:** resources (supporting). **Mitsuhiro Fujishiro:** supervision (equal).

## Ethics Statement

This retrospective study was conducted in accordance with the ethical guidelines for epidemiological research of the Japanese Ministry of Education, Culture, Sports, Science, and Technology and the Ministry of Health, Labor, and Welfare. This study was included in a comprehensive protocol of retrospective studies conducted in the Department of Gastroenterology of the University of Tokyo Hospital and was approved by the University of Tokyo Medical Research Center Ethics Committee (approval number 2058). The need for informed consent was waived by the University of Tokyo Medical Research Center Ethics Committee. The following statements were posted on a website (http://gastro.m.u‐tokyo.ac.jp/patient/clinicalresearch.html) and participants who did not agree to the use of their clinical data could request to be excluded.

## Conflicts of Interest

The authors declare no conflicts of interest.

## Supporting information


**Supplementary Figure 1.** Treatment schedule and imaging evaluation protocol at our institution. The upper row represents the Atezo/Bev group, whereas the lower row represents the LEN group. The image evaluation dates for both groups closely matched at weeks 8 and 16. Atezo/Bev, atezolizumab plus bevacizumab; LEN, lenvatinib.

**Supplementary Table 1.** Adverse events (≥ 10%).*

## Data Availability

The data that support the findings of this study are not publicly available due to ethical considerations. However, they are available from the corresponding author upon reasonable request.
