# Supplementary figures and images for: Anti‐Angiogenic Agents for Advanced Hepatocellular Carcinoma Induce Liver Atrophy
Source: Cancer Med. 2025 Jul 25;14(15):e71066. doi: 10.1002/cam4.71066 (PMC12290651; doi:10.1002/cam4.71066)

Supplementary Figure 1

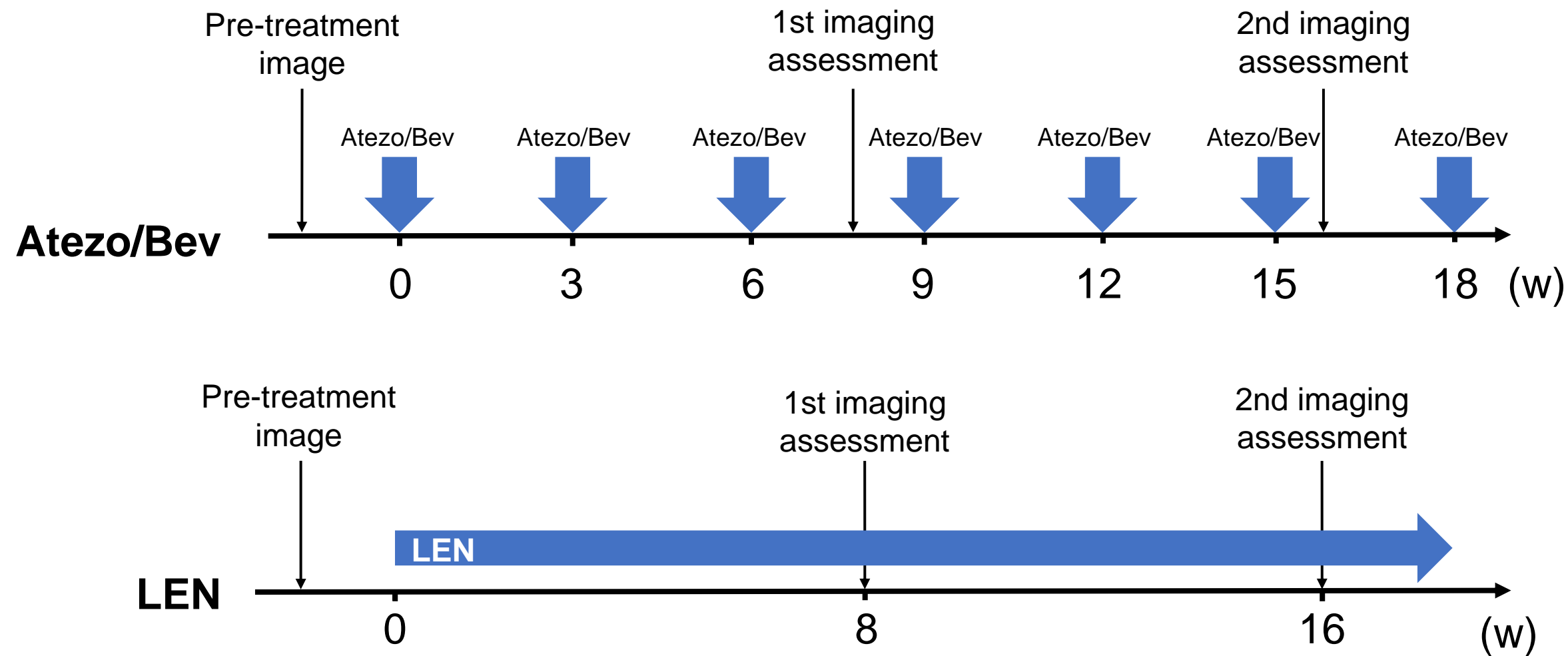

Supplement: Supplementary file 1 — Supplementary Figure 1. Treatment schedule and imaging evaluation protocol at our institution. The upper row represents the Atezo/Bev group, whereas the lower row represents the LEN group. The image evaluation dates for both groups closely matched at weeks 8 and 16. Atezo/Bev, atezolizumab plus bevacizumab; LEN, lenvatinib. [file CAM4-14-e71066-s002.pdf]
