# Supplementary material for: Anti‐Angiogenic Agents for Advanced Hepatocellular Carcinoma Induce Liver Atrophy
Source: Cancer Med. 2025 Jul 25;14(15):e71066. doi: 10.1002/cam4.71066 (PMC12290651; doi:10.1002/cam4.71066)
Supplement: Supplementary file 2 — Supplementary Table 1. Adverse events (≥ 10%).* [file CAM4-14-e71066-s001.docx]

**Supplementary Table 1. Adverse events (≥10%).^*^**

|  | **Atezo/Bev Group** | | **LEN Group** | |
| --- | --- | --- | --- | --- |
|  | N = 40 | | N = 33 | |
|  | **any grade** | **Grade ≥3** | **any grade** | **Grade ≥3** |
| Hypertension | 7 (17.5) | 1 (2.5) | 4 (12.1) | 1 (3.0) |
| Fatigue | 11 (27.5) | 2 (5.0) | 13 (39.4) | 3 (9.1) |
| Proteinuria | 11 (27.5) | 5 (12.5) | 12 (36.4) | 7 (21.2) |
| Appetite loss | 4 (10.0) | 1 (2.5) | 11 (33.3) | 3 (9.1) |
| Hypothyroidism | 4 (10.0) | 0 | 5 (15.2) | 2 (6.1) |
| Liver dysfunction | 4 (10.0) | 1 (2.5) | 3 (9.1) | 3 (9.1) |
| Diarrhea | 1 (2.5) | 0 | 3 (9.1) | 1 (3.0) |
| Hoarseness | 7 (17.5) | 0 | 2 (6.1) | 0 |
| Bleeding | 6 (15.0) | 3 (7.5) | 0 | 0 |
| Rash | 7 (17.5) | 0 | 0 | 0 |
| Palmar-plantar erythrodysesthesia syndrome | 2 (5.0) | 0 | 7 (21.2) | 4 (12.1) |
| Decreased platelet count | 5 (12.5) | 0 | 0 | 0 |

^*^ Adverse events that occurred in more than 10% of either treatment groups are listed. Data are presented as n (%).

Atezo/Bev, atezolizumab plus bevacizumab; LEN, lenvatinib.
